# Dissecting Causal Links Between Gut Microbiota, Inflammatory Cytokines, and Parkinson's Disease: A Mendelian Randomization Study

**DOI:** 10.1002/brb3.70169

**Published:** 2024-11-28

**Authors:** Ma Caiyun, Wen Hebao, Ye Wenhao, Liu Changqing, Li Changqing, Zhao Xiaojiang

**Affiliations:** ^1^ Anhui Engineering Research Center for Neural Regeneration Technology and Medical New Materials Bengbu Medical University Bengbu China; ^2^ Department of Physical Education and Arts Bengbu Medical University Bengbu China

**Keywords:** gut microbiota, inflammatory cytokines, Mendelian randomization, Parkinson's disease

## Abstract

**Background:**

The association between gut microbiota (GM) and Parkinson's disease (PD) has been well established, but whether there is a causal relationship between the two and whether inflammatory cytokines (ICs) act as mediators remain unclear.

**Methods:**

We utilized the summary databases of large‐scale genome‐wide association studies (GWAS) conducting Mendelian randomization (MR) analyses to investigate the causal relationships between GM, ICs, and PD. The inverse‐variance weighted (IVW) method was primarily used to identify GM and ICs associated with PD and to examine the mediating role of ICs, supplemented by MR Egger and weighted median.

**Results:**

Through MR analysis, we identified three positive causal relationships and six negative causal relationships between GM and PD. Additionally, there were three positive associations and five negative associations between ICs and PD. However, after adjusting for FDR, none of these associations were significant. In reverse MR analysis, we also found causal relationships between PD and various GM and ICs. Further, two‐step MR analysis indicated that the negative impact of phylum Actinobacteria on PD may be mediated through Fms‐related tyrosine kinase 3 ligand levels.

**Conclusion:**

This study strengthens the link between GM and the risk of PD, while also revealing the potential mediating role of ICs in the causal relationships between these factors.

## Graphical abstract

Step 1 and Step 2 represent the bidirectional causal relationships between gut microbiota, inflammatory cytokines, and Parkinson's disease. Step 3 represents the mediating analysis of inflammatory cytokines in the pathway from gut microbiota to Parkinson's disease.

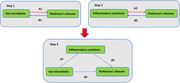

## 1 Introduction

Parkinson's disease (PD) is the second most common neurodegenerative disease after Alzheimer's disease (Ortelli et al. 2023). Globally, over 8.5 million individuals suffer from PD, with its onset closely associated with aging (Ratan et al. 2023). It is projected that by 2030, half of the global PD patients will be Chinese (Chen and Geng 2023; Wang and Huang 2023). Therefore, monitoring the prevalence of PD and exploring new treatment approaches has become crucial. PD is characterized by both motor and nonmotor symptoms, severely impacting patients' quality of life. Studies have found that the majority of PD patients experience gastrointestinal symptoms such as nausea, constipation, and delayed gastric emptying (Balestrino and Schapira 2020). Approximately 80% of PD patients suffer from constipation, and these symptoms often precede PD motor symptoms by an average of 10 years or more (Yang et al. 2019).

The human gut microbiota (GM) is approximately 10^13^ to 10^14^ microbes, with the total number of genes in the gut microbes being more than 150 times that of the human genome (Alkasir et al. 2017). The microbiota–gut–brain axis (MGBA) is a bidirectional communication system that integrates brain and gut functions, where GM plays a crucial role (Karakan et al. 2021; Rao et al. 2021). Recent studies have increasingly found that dysbiosis of the GM is highly associated with PD onset and progression and may promote PD development through the MGBA. GM communicates with the brain through the nervous, immune, and neurotransmitters.

Inflammatory response is a crucial defense mechanism of the body's immune system. In cases of GM dysbiosis, neurotoxic molecules such as lipopolysaccharides (LPS) penetrate the damaged gut barrier, coming into contact with immune cells and enteric neurons. The cytokines secreted by immune cells interact with cortisol, participating in communication between the gut and the brain. In addition, α‐synuclein (α‐syn) produced by enteric neurons can retrogradely travel along the vagus nerve to the brain, activating glial cells, inducing neuroinflammation, and damaging dopaminergic neurons. GM and inflammatory cytokines (ICs) can both influence the progression of PD. We hypothesize that ICs may act as mediators in the pathway from GM to PD.

Mendelian randomization (MR) is an analytical technique used in epidemiological studies to assess causal inferences. It utilizes genetic variants strongly associated with exposure factors as instrumental variables (IVs) to evaluate the causal relationship between exposure and outcome. This method overcomes potential confounding and reverses causality, making the inference of associations more reliable.

In this study, we conducted a two‐sample, bidirectional, two‐step MR analysis method to explore the causal relationship between GM, ICs, and PD. We investigated whether ICs act as mediators in the pathway from GM to PD. Additionally, through reverse causality analysis, we examined whether genetic susceptibility to PD affects GM and ICs.

## 2 Materials and Methods

### 2.1 Study Design

**FIGURE 1:**
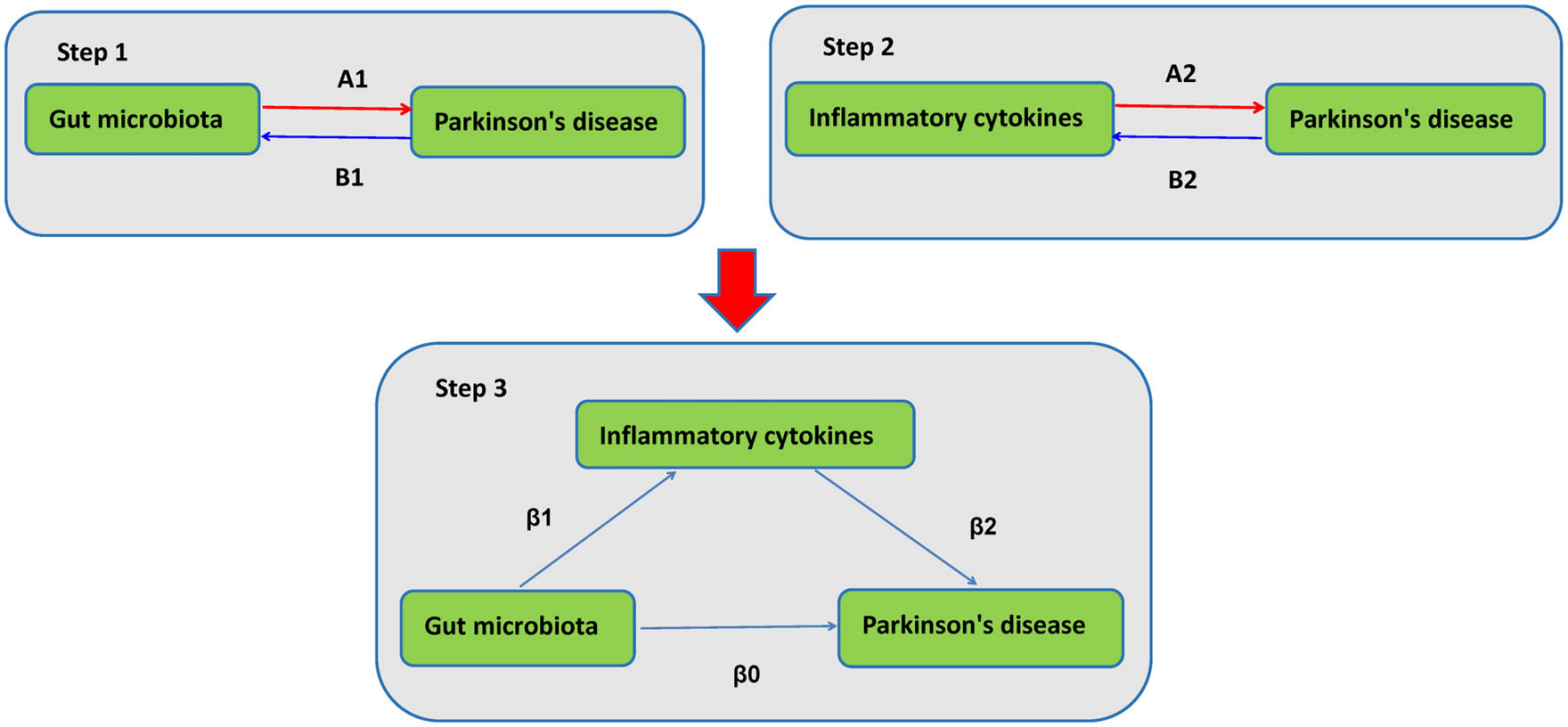
Flow chart showing the study design. Step 1: A1 represents the causal effects of GM on PD; B1 represents the reverse causal effects of GM on PD. Step 2: A2 represents the causal effects of ICs on PD; B2 represents the reverse causal effects between ICs on PD. Step 3: represents the mediating analysis of ICs in the pathway from GM to PD: β0 was the total effect of GM on PD; β1 represents the causal effects of GM on ICs; β2 represents the causal effects of ICs on PD. The mediation effect was computed as the product of “β1” and “β2” (β1 × β1), and the mediation proportion was calculated as the ratio of the mediation effect product to total effects [(β1 × β1)/β0]. Abbreviations: GM = gut microbiota; ICs = inflammatory cytokines; PD = Parkinson's disease.

Our study investigated genetic associations between GM, ICs, and PD using publicly available genome‐wide association studies (GWAS) data. First, we employed bidirectional two‐sample MR analyses to reveal the bidirectional causal relationships between GM, ICs, and PD. Subsequently, to explore the potential mediating role of ICs between GM and PD, we conducted a two‐step MR analysis. This involved assessing the causal impact of GM on ICs, followed by examining the causal impact of these ICs on PD. We employed multivariate Mendelian randomization (MVMR) to evaluate the direct effect of GM on PD, considering potential mediators. Single nucleotide polymorphisms (SNPs) are defined as IVs in the context of MR, which relies on three fundamental assumptions: (1) the IVs are strongly correlated with the exposure factors, (2) the IVs are not influenced by confounding factors, and (3) the IVs do not directly impact the outcome but rather exert their effects solely through the exposure (Davey Smith and Hemani 2014). This study was conducted according to STROBE‐MR guidelines [11]. Figure 1 illustrates the study's design schematically.

### 2.2 Data Source

A summary of the GWAS statistics used for statistical analysis can be found in Table 1. This study defines the abundance of GM and IVs as exposure factors and PD as the outcome factor. We obtained GM data from the extensive analysis conducted by the MiBioGen consortium (Kurilshikov et al. 2021). This dataset integrates genetic information from 24 cohorts, encompassing 18,340 individuals from 11 countries, with the majority of participants being of European ancestry (Kurilshikov et al. 2021). The systematic compilation process resulted in a dataset containing 196 bacterial taxa, providing a comprehensive foundation for in‐depth analysis. GWAS data covering 91 IVs were gathered from 11 cohorts, which involved 14,824 participants of European descent [13]. The summary data for the results section comes from the FinnGen Biobank (Round 11) (https://www.finngen.fi/en/access_results). The Parkinson's GWAS sample size in FinnGen is 453,733 individuals.

**TABLE 1:** GWAS summary data sources used in MR analyses.

Phenotype	GWAS	Sample size
Gut microbiota	Kurilshikov et al. 2021	18,340
Inflammatory cytokines	Zhao et al., 2023	14,824
Parkinson	FinnGen R11	453,733

Abbreviation: GWAS = genome‐wide association study.

### 2.3 IV Selection and Data Harmonization

We applied a series of filtering criteria to select eligible genetic instruments. Initially, we planned to use a significance threshold of *p* < 5 × 10^−8^ to select IVs for GM and ICs. Due to the very small number of eligible IVs, based on previous studies (Kurilshikov et al. 2021; Zhong et al. 2023), we adopted more lenient statistical thresholds. Ultimately, we applied a significance threshold of *p* < 1 × 10^−5^ to select IVs related to GM and *p* < 5 × 10^−6^ to select IVs related to ICs. Precise criteria were defined for the selection of SNPs, employing a threshold of *R* ^2^ < 0.001 and kb = 10,000 to eliminate SNPs within a 10 Mb region exhibiting linkage disequilibrium with the most significant SNP (*R* ^2^ > 0.001). This methodology aided in the elimination of SNPs susceptible to linkage disequilibrium influences. Furthermore, SNPs with palindromic or ambiguous characteristics were systematically excluded from IVs utilized in the MR analysis. *F*‐statistics were computed using *F* = β^2^ / SE^2^ [15] in order to prevent weak IVs.

### 2.4 Statistical Analyses

First, we conducted comprehensive bidirectional two‐sample MR analyses to evaluate the bidirectional causal relationships between GM, ICs, and PD. Multiple methods were employed in this study, with the inverse‐variance weighted (IVW) method serving as the primary approach and the weighted median method and MR Egger method as secondary approaches. Odds ratios (OR) and their corresponding 95% confidence intervals (95% CI) were used to present the MR results. Statistical significance was determined by a *p*‐value of less than 0.05 for the IVW approach, while the other two methods were in agreement with the IVW method. False discovery rate (FDR) correction was applied using the *q*‐value method. Results were considered statistically significant when FDR *q* < 0.1 but suggestive of an association when *q* ≥ 0.1 and *p* < 0.05. Next, we conducted MVMR analysis to determine the mediating role of ICs in the relationship between GM and PD.

### 2.5 Sensitivity Analysis

Multiple sensitivity analyses and statistical tests were employed to evaluate the soundness of assumptions. A Cochran *Q* test was utilized, with a heterogeneity *p*‐value of less than 0.05 indicating the presence of heterogeneity. In cases of significant heterogeneity, a random effects model was applied, while a fixed effects model was used otherwise. MR‐PRESSO global test and MR‐Egger intercept were conducted to detect outliers and horizontal pleiotropy. The MR‐Egger intercept signifies the average pleiotropic effect, and a *p*‐value below 0.05 indicates the generation of reliable pleiotropic MR estimates. All statistical analyses were performed using R and R‐studio (version 4.0.3). MR analyses were performed using the TwosampleMR, MR‐PRESSO (version 1.0), and MVMR (version 0.4) R packages.

### 2.6 Ethical Statement

Participants consented to the GWAS as per the original protocols, and all ethical approvals were obtained by the original authors.

## 3 Results

### 3.1 Selection of IVs

Through screening and analysis, we found that nine different GM and eight different ICs have potential causal relationships with PD. Since the *F*‐statistics of the IVs we selected are all above 10, these data indicate that there are no weak IVs. The results can be found in Tables S1 and S2.

### 3.2 Causal Association Between GM With PD

**FIGURE 2:**
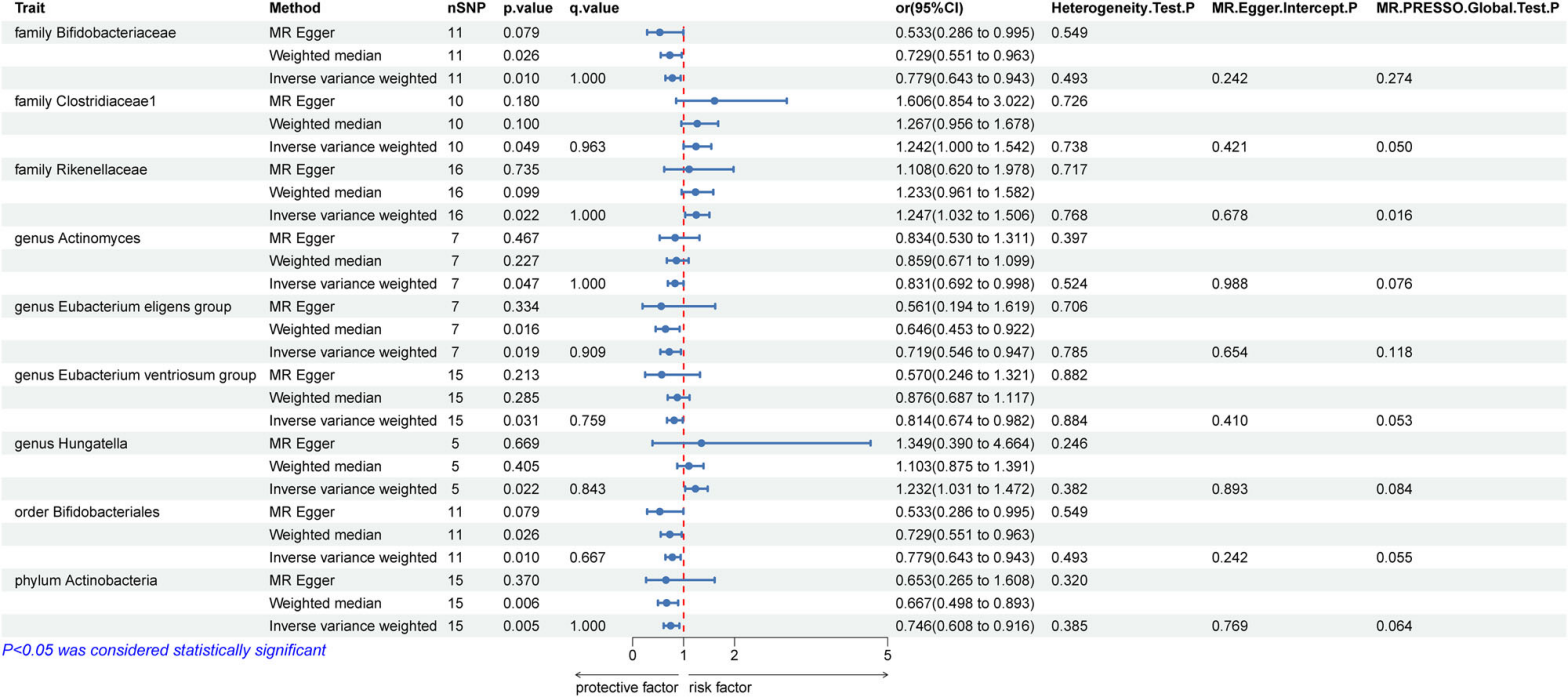
MR estimates for the association between GM and PD. Abbreviations: GM = gut microbiota; IVW = inverse‐variance weighted; nSNP = number of single‐nucleotide polymorphism; PD = Parkinson's disease.

As shown in Figure 2, our analysis identified potential causal relationships between different GM and PD. Among these nine microbiota, three (family Clostridiaceae1, family Rikenellaceae, genus *Hungatella*) increase the risk of PD, while six (family Bifidobacteriaceae, genus *Actinomyces*, genus *Eubacterium eligens* group, genus *Eubacterium ventriosum* group, order Bifidobacteriales, phylum Actinobacteria) reduce the risk of PD. The IVW analysis results for these nine types of GM are as follows: family Clostridiaceae1 (OR = 1.242; 95% CI = 1.000–1.542; *p* = 0.049; *q* = 0.963), family Rikenellaceae (OR = 1.247; 95% CI = 1.032–1.506; *p* = 0.022; *q* = 1.000), genus *Hungatella* (OR = 1.232; 95% CI = 1.031–1.472; *p* = 0.022; *q* = 0.843), family Bifidobacteriaceae (OR = 0.779; 95% CI = 0.643–0.943; *p* = 0.010; *q* = 1.000), genus *Actinomyces* (OR = 0.831; 95% CI = 0.692–0.998; *p* = 0.047; *q* = 1.000), genus *E. eligens* group (OR = 0.719; 95% CI = 0.546–0.947; *p* = 0.019; *q* = 0.909), genus *E. ventriosum* group (OR = 0.814; 95% CI = 0.674–0.982; *p* = 0.031; *q* = 0.759), order Bifidobacteriales (OR = 0.779; 95% CI = 0.643–0.943; *p* = 0.010; *q* = 0.667), and phylum Actinobacteria (OR = 0.746; 95% CI = 0.608–0.916; *p* = 0.005; *q* = 1.000). However, after applying the FDR correction, no bacterial taxa were found to be significantly associated.

### 3.3 Causal Association Between ICs With PD

**FIGURE 3:**
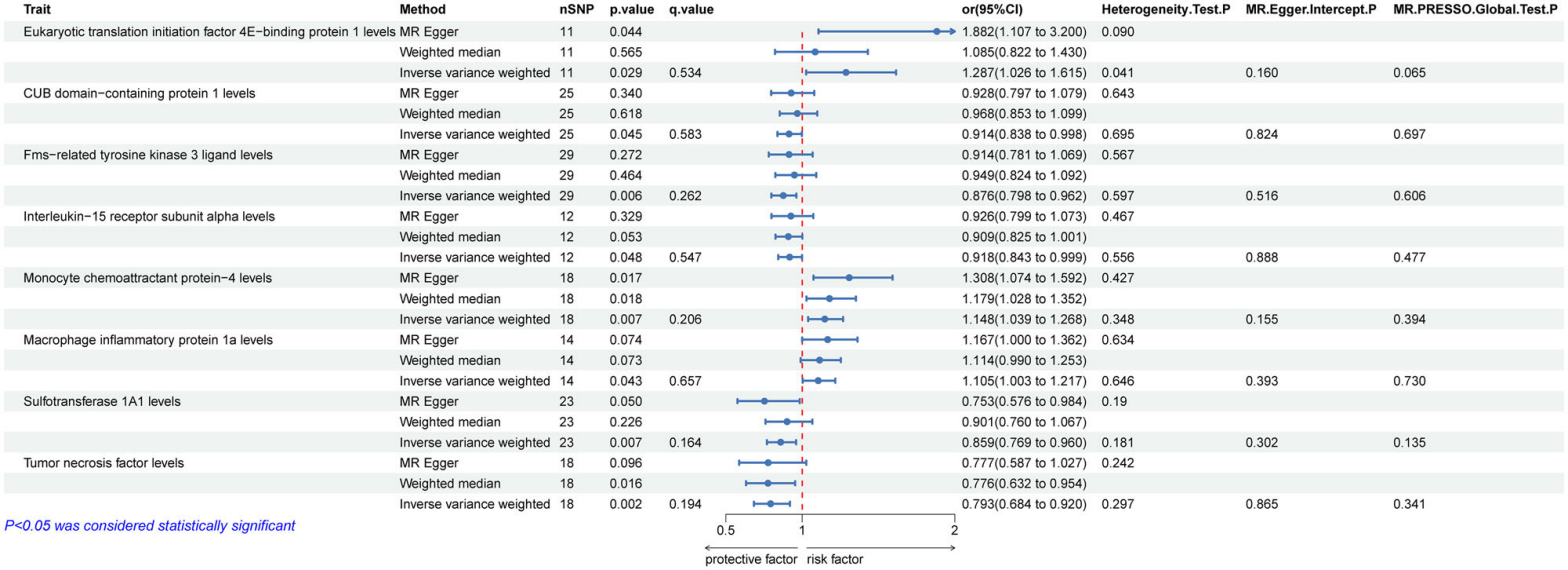
MR estimates for the association between ICs and PD. Abbreviations: ICs = inflammatory cytokines; IVW = inverse‐variance weighted; nSNP = number of single‐nucleotide polymorphisms; PD = Parkinson's disease.

We conducted a two‐sample MR analysis to assess the relationship between eight types of ICs and the risk of PD. Eukaryotic translation initiation factor 4E−binding protein 1 levels (OR = 1.287; 95% CI = 1.026–1.615; *p* = 0.029; *q* = 0.534), Monocyte chemoattractant protein−4 levels (OR = 1.148; 95% CI = 1.039–1.268; *p* = 0.007; *q* = 0.206), and Monocyte inflammatory protein 1a levels (OR = 1.015; 95% CI = 1.003–1.217; *p* = 0.043; *q* = 0.657) were positively associated with PD, while the CUB domain−containing protein 1 levels (OR = 0.968; 95% CI = 0.853–1.099; *p* = 0.045; *q* = 0.583), Fms‐related tyrosine kinase 3 ligand levels (OR = 0.876; 95% CI = 0.798–0.962; *p* = 0.006; *q* = 0.262), Interleukin‐15 receptor subunit alpha levels (OR = 0.918; 95% CI = 0.843–0.999; *p* = 0.048; *q* = 0.547), Sulfotransferase 1A1 levels (OR = 0.859; 95% CI = 0.769–0.960; *p* = 0.007; *q* = 0.164), and tumor necrosis factor levels (OR = 0.793; 95% CI = 0.684–0.920; *p* = 0.002; *q* = 0.194) were negatively associated with PD (Figure 3). Subsequent to the implementation of the FDR correction for multiple comparisons, the aforementioned associations did not demonstrate statistical significance.

### 3.4 Reverse MR Analysis and Mediation Analysis

**FIGURE 4:**
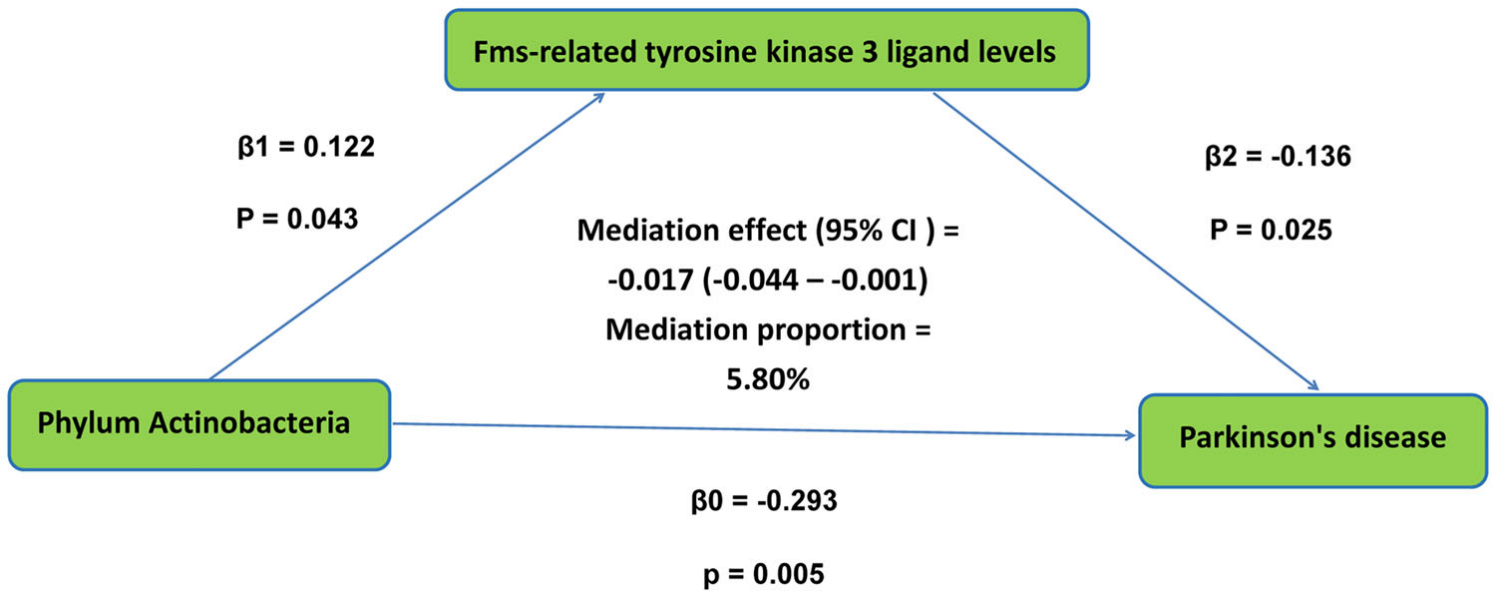
The Fms‐related tyrosine kinase 3 ligand levels mediated the causal effect of phylum Actinobacteria on PD. The β1 value between the phylum Actinobacteria and Fms‐related tyrosine kinase 3 ligand levels and the β2 value between Fms‐related tyrosine kinase 3 ligand levels and Parkinson are MR estimates using the inverse‐variance weighted method; PD = Parkinson's disease.

For reverse MR analysis, as shown in Tables S3 and S4, we found three causal associations between PD and GM and three associations between PD and ICs. We used two‐sample MR to assess the causal relationships between GM and ICs that are both causally associated with PD and to screen for correlations between GM and ICs (Table S5). To explore potential mediators, we conducted mediation analysis using MVMR to identify potential mediators (Figure 4). Specifically, Fms‐related tyrosine kinase 3 ligand levels mediated the causal relationship between phylum Actinobacteria and PD, with a mediation proportion of 5.80%.

### 3.5 Sensitivity Analysis

To assess the potential impact of pleiotropy on the causal effect estimates, several sensitivity analyses were conducted. Both the IVW (*p* > 0.05) and MR Egger analysis results (*p* > 0.05) showed no heterogeneity. Additionally, the MR‐Egger regression intercept test (*p* > 0.05) and MR‐PRESSO global test (*p* > 0.05) provided no evidence of pleiotropy (Figures 2 and 3).

## 4 Discussion

Currently, PD treatment primarily relies on medication, mainly using levodopa or dopamine receptor agonists to alleviate symptoms (Cong et al. 2022; Liu and Le 2020).

Recent studies have increasingly found that dysbiosis of GM is highly correlated with the onset and progression of PD and may promote PD progression through the MGBA (Agirman, Yu, and Hsiao 2021; Varesi et al. 2022). Challis et al. (2020) found that injecting α‐syn‐preformed fibrils into the duodenal wall of aged mice induced the formation of phosphorylated α‐syn, which then spread to the brain, causing gastrointestinal dysfunction and motor impairments. Bhattarai et al. (2021) administered rotenone orally to both germ‐free (GF) and conventionally raised (CR) mice for 6 consecutive weeks. They found that chronic rotenone administration increased gut permeability and reduced motor strength and coordination in CR mice but did not alter gut permeability or cause motor deficits in GF mice. These results suggest that PD pathology may originate in the gut, with GM dysbiosis being associated with increased gut permeability and motor dysfunction.

In recent years, understanding the relationship between GM and PD has led to a focus on treating PD by modulating the composition of GM. Currently, there is no unified standard for a healthy gut. Most studies suggest that the more diverse and stable the GM, the healthier the organism. Abnormal changes in the composition and diversity of GM can lead to dysbiosis, potentially causing abnormal changes in microbial metabolites, such as a decrease in beneficial substances like short‐chain fatty acids (SCFAs) and an increase in harmful substances like LPS. These toxic substances may retrogradely travel to the central nervous system via the MGBA, impacting the onset and progression of PD. Additionally, neurotoxic molecules can penetrate the blood‐brain barrier through peripheral circulation, leading to dopaminergic neurodegeneration (Fan, Sheng, and Zhang 2022; Han et al. 2022). A study has confirmed that transplantation of fecal microbiota (FMT) from a healthy donor to patients with mild to moderate PD via nasojejunal administration resulted in improvements in objective indicators of constipation and motor symptoms, and this trial provides evidence for gut microbe‐targeted therapy for PD (Bruggeman et al. 2024). Another researcher found that FMT was safe. Still, due to the different donors, changes in the microbiota were more pronounced in PD patients after FMT, with a higher frequency of remission of ecological dysregulation with the placebo group. Therefore, given the composition of the donor microbiota and the impact of dysbiosis conversion on motor and nonmotor symptoms in PD patients, there is a need for improved FMT approaches or bowel cleansing in the future (Scheperjans et al. 2024).

Changes in the composition of GM and gut permeability in PD patients can activate Toll‐like receptors (TLRs) and innate immune responses, enhancing the inflammatory effect of α‐syn and leading to its misfolding (Caputi and Giron 2018; Mou et al. 2022). Misfolded α‐syn binds to TLR2 on microglia, activating downstream pathways involving myeloid differentiation factor 88 (MyD88) and nuclear factor kappa‐B (NF‐κB), which trigger the production of TNF‐α and IL‐1β, exacerbating neuroinflammatory damage (Xia et al. 2021). Therefore, changes in GM may lead to neuroinflammation, and inhibiting neuroinflammation associated with glial cell activation is a potential therapeutic target for PD.

Our study investigates the causal relationships between nine GM, eight immune cytokines, and PD. The results indicated that for PD, some GM are risk factors while others are protective factors. This study determined the beneficial or harmful effects of GM on PD by genetic data and MR analysis and also revealed the mediating role of ICs in the causal relationships between GM and PD. Among nine types of GM in our present study, six of them (family Bifidobacteriaceae, genus *Actinomyces*, genus *E. eligens* group, genus *E. ventriosum* group, order Bifidobacteriales, phylum Actinobacteria) were inversely associated with PD, whereas three of them (family Clostridiaceae1, family Rikenellaceae, genus *Hungatella*) were positively associated with PD. Due to the complexity of the GM, observational studies are insufficient to comprehensively summarize its impact on PD. These findings offer a novel perspective on understanding the molecular mechanisms and provide potential targets for future therapeutic strategies.

Our mediation analysis has obtained genetic evidence related to GM, ICs, and PD. This study appears to be the first to establish a direct link between phylum Actinobacteria, Fms‐related tyrosine kinase 3 ligand levels, and PD. Although previous studies suggested a potential association between them, none has definitively demonstrated that Fms‐related tyrosine kinase 3 ligand levels mediate the causal relationship between phylum Actinobacteria and PD. Chin et al. reported that dysbiosis of the GM in patients with PD can trigger immune activation, leading to PD by stimulating ICs (Chin et al., 2019).

This study has several advantages that can guide future research on PD. Firstly, the study is the first to use MR analysis to investigate the causal relationships between GM, ICs, and PD. Our comprehensive analysis of 196 different types of GM and 91 types of ICs highlights this unique contribution. Using the MR design, this study effectively reduces the problems of reverse causality and residual confounding. In addition, we conducted sensitivity analyses to eliminate potential impacts of genetic pleiotropy, thus enhancing the validity of our causal inferences. Secondly, we examined the potential mediating role of ICs in these causal relationships through MVMR analysis. Lastly, we employed FDR correction for multiple comparisons. However, this study also has limitations. Firstly, since the sample is predominantly of European ancestry, our findings may not be applicable to other populations, potentially failing to accurately reflect the genetic and lifestyle diversity affecting GM in different groups. Secondly, it is also important to recognize that our findings did not survive the strict threshold of the FDR correction. However, applying such a stringent correction has the potential to obscure biologically meaningful findings. Finally, while we explored how ICs mediate the relationship between GM and PD, the mechanisms by which GM affects PD pathogenesis need further study. Therefore, more basic experimental and clinical studies are needed to establish the causal relationship between GM and PD and the long‐term effects of GM intervention.

## 5 Conclusion

To our knowledge, this is the first comprehensive study evaluating the causal relationships between GM, ICs, and PD. Our results highlight the necessity of exploring the mechanisms underlying the interactions between GM, ICs, and PD. Furthermore, our findings reveal the importance of elucidating the potential mechanisms mediated by ICs between GM and PD. We believe these results provide new insights into the immunotherapy and GM‐targeted interventions for PD.

## Author Contributions

**Ma Caiyun**: conceptualization, validation, investigation, writing–original draft. **Wen Hebao**: writing–original draft, project administration. **Ye Wenhao**: validation, data curation, visualization. **Liu Changqing**: software, formal analysis, visualization. **Zhao Xiaojiang**: resources, conceptualization, writing–original draft. **Li Changqing**: methodology, writing–original draft.

## Ethics Statement

Participants consented to the GWAS as per the original protocols, and the original authors were responsible for obtaining all ethical approvals.

## Conflicts of Interest

The authors declare no conflicts of interest.

### Peer Review

The peer review history for this article is available at https://publons.com/publon/10.1002/brb3.70169

## Supporting information



Supplementary Materials.

## Data Availability

The datasets that were analyzed in this study are not publicly available. Further inquiries can be directed to the corresponding author.
